# Peer Review of “Assessing the Limitations of Large Language Models in Clinical Practice Guideline–Concordant Treatment Decision-Making on Real-World Data: Retrospective Study”

**DOI:** 10.2196/84174

**Published:** 2025-11-03

**Authors:** Andrej Novak

**Affiliations:** 1 University of Zagreb, Ul. Radoslava Cimermana 88, Zagreb, Croatia, 385 1 4564 111

**Keywords:** large language model, foundation model, reasoning model, treatment decision-making, aortic stenosis, clinical practice guidelines, medical data processing

*This is the peer-review report for “Assessing the Limitations of Large Language Models in Clinical Practice Guideline–Concordant Treatment Decision-Making on Real-World Data: Retrospective Study.”*

## Round 1 Review

The authors of this paper [1] set out to determine whether modern large language models (LLMs) can make treatment decisions for severe aortic stenosis based on uncurated, free-text clinical data in routine practice. This question addresses a significant gap in the literature: while earlier work demonstrated that LLMs could agree with expert tumor boards or heart teams when provided with highly structured or preprocessed information, the realities of clinical documentation—discharge summaries, imaging reports, and free-text notes—remain unstructured and noisy. It seems that even top LLMs fail to deliver reliable, unbiased treatment recommendations from raw clinical text. They perform well only with expert-crafted summaries and embedded guidelines, highlighting that data representation (and prompt engineering) is key.

Methods: The authors should be commended for the exceptionally detailed Methods section, which carefully notes subtleties such as each model’s maximum context. Their inclusion of a simple non-LLM reference model alongside a broad spectrum of open- and closed-source LLMs represents thoughtful experimental design, and their 4-arm RAW→RAW+→SUM→SUM+ framework neatly isolates the impact of data representation and guideline context.

Analysis and Results: Using Cohen κ to assess agreement on a binary decision task is appropriate; when paired with accuracy, it gives a fuller picture than raw percentages alone. Supplementing κ with intraclass correlation coefficients (ICCs) and normalized Shannon entropy to gauge reliability across repeated runs is also sound. The results themselves are compelling. Across 9 models, κ values on raw text ranged from slight negative agreement up to only fair (–0.47 to 0.22), and ICCs were poor, demonstrating that without curated input, even leading LLMs can not reliably distinguish surgical aortic valve replacement from transcatheter aortic valve replacement. When expert summaries plus guideline text were provided, κ jumped into the moderate‐substantial range (up to 0.63) and ICCs reached good–excellent levels. That consistent, monotonic improvement from RAW→RAW+→SUM→SUM+ (replicated across open and closed models) makes a strong, convincing case that data representation, not just model capability, drives performance.

That said, the retrospective, single-center design with only 80 cases further constrains generalizability; patient populations and documentation styles vary widely across institutions. The way indeterminate recommendations were handled in metrics (counted as “wrong” for κ and accuracy, but excluded from bias calculations) may also skew the interpretation of model caution versus error. Finally (as noted in the Limitations), on a philosophical level—treating heart-team decisions, which are themselves subjective, as infallible ground truth risks overstating LLM shortcomings.

Beyond the major strengths and limitations previously discussed, I have identified several minor points that would further strengthen the manuscript:

1. The format and provenance of the SUM (“case summary”) reports require clearer specification. Although the authors note these summaries were “manually generated,” it would be helpful to state whether they followed a standardized template, who exactly drafted them (eg, experienced cardiologists, research assistants), and which elements of the Heart Team protocol they distilled into each summary.

2. The authors report that the original medical documents were saved as PDFs and later converted to plain text. It would be helpful to clarify this process to avoid confusion, since LLMs accessed via chat interfaces or application programming interfaces often struggle with PDF inputs or text embedded in images, treating them differently from pure text. A brief discussion acknowledging this limitation—and explaining how PDF parsing was handled or validated—would help readers assess real-world applicability.

3. Raw inputs (PDFs and summaries) were provided in German (except for BioGPT, which required translation to English). A comment in the Discussion about how model performance can vary by input language—perhaps citing studies that showed different results in Polish versus English—would contextualize the findings for non-English clinical settings:

- Rosoł M, Gąsior JS, Łaba J, Korzeniewski K, Młyńczak M. Evaluation of the performance of GPT-3.5 and GPT-4 on the Polish Medical Final Examination. *Sci Rep*. 2023;13(1):20512.

4. The Discussion section feels comparatively weak and could be strengthened by broader literature coverage. For instance, a brief discussion of input formats—pure text versus multimodal inputs—would be valuable, especially given the inclusion of GPT-4o, which handles images. Preliminary studies in this area include:

- Günay et al. Comparison of emergency medicine specialist, cardiologist, and ChatGPT in electrocardiography assessment. *Am J Emerg Med*. 2024 Jun;80:51-60.
- Zeljkovic et al. Beyond text: the impact of clinical context on GPT-4’s 12-lead electrocardiogram interpretation accuracy. *Canadian J Cardiol*. 2025 Jul;41(7):1406-1414.

These compare electrocardiogram interpretation with and without accompanying clinical context and demonstrate the importance of textual input alongside images.

It would also be helpful to reference work showing that, despite similar hallucination tendencies, LLMs perform strongly on standardized exams, for example:

- Gilson et al. How does ChatGPT perform on the USMLE? Implications for medical education and knowledge assessment. *JMIR Med Educ*. 2023 Feb 8;9:e45312.
- Novak et al. The pulse of artificial intelligence in cardiology: evaluating state-of-the-art LLMs for clinical cardiology. *medRxiv*. Preprint posted online on January 30, 2024.

These additions could situate the findings within a broader context of multimodal and high-stakes assessment.

 5. As an exploratory aside, it would be interesting to evaluate how the newest reasoning-focused models (eg, “o3” or “o4”) perform on this task. Although this is likely beyond the current scope, including a sentence to that effect in the manuscript’s Limitations section could guide future research.

6. For consistency and precision, when describing model access in the “Large Language Models” section (and elsewhere in the text), the manuscript should explicitly cite the exact supplementary tables or materials (eg, “see Table S1 for model details and context sizes”) rather than referring generically to “the Supplementary.”

 7. In the Statistical Methods subsection, rather than stating that nonnormally distributed data were compared using the Mann-Whitney *U* test “for nonnormally distributed continuous variables,” the phrasing could be tightened to “for variables departing from normality” or “for variables not following a normal distribution” to align with standard statistical terminology.

## Abbreviations

ICC: intraclass correlation coefficient
LLM: large language model
